# Dual-Specificity Phosphatase 6 Deficiency Attenuates Arterial-Injury-Induced Intimal Hyperplasia in Mice

**DOI:** 10.3390/ijms242417136

**Published:** 2023-12-05

**Authors:** Candra D. Hamdin, Meng-Ling Wu, Chen-Mei Chen, Yen-Chun Ho, Wei-Cheng Jiang, Pei-Yu Gung, Hua-Hui Ho, Huai-Chia Chuang, Tse-Hua Tan, Shaw-Fang Yet

**Affiliations:** 1 Institute of Cellular and System Medicine, National Health Research Institutes, Zhunan 350401, Taiwan; cdh@nhri.edu.tw (C.D.H.); pygung@nhri.edu.tw (P.-Y.G.); legend8240@nhri.edu.tw (H.-H.H.); 2 National Health Research Institutes and Department of Life Sciences, National Central University Joint Ph.D. Program in Biomedicine, Zhongli District, Taoyuan 320317, Taiwan; 3 Cardiovascular Biology Research Program, Oklahoma Medical Research Foundation, Oklahoma City, OK 73104, USA; melinda-wu@omrf.org (M.-L.W.); yenchun-ho@omrf.org (Y.-C.H.); 4 Department of Anatomy and Cell Biology, College of Medicine, National Yang Ming Chiao Tung University, Taipei 112304, Taiwan; wcjiang@nycu.edu.tw; 5 Immunology Research Center, National Health Research Institutes, Zhunan 350401, Taiwan; cinth@nhri.org.tw (H.-C.C.); ttan@nhri.edu.tw (T.-H.T.); 6 Graduate Institute of Biomedical Sciences, China Medical University, Taichung 404328, Taiwan

**Keywords:** DUSP6, vascular remodeling, VSMC, proliferation, migration

## Abstract

In response to injury, vascular smooth muscle cells (VSMCs) of the arterial wall dedifferentiate into a proliferative and migratory phenotype, leading to intimal hyperplasia. The ERK1/2 pathway participates in cellular proliferation and migration, while dual-specificity phosphatase 6 (DUSP6, also named MKP3) can dephosphorylate activated ERK1/2. We showed that DUSP6 was expressed in low baseline levels in normal arteries; however, arterial injury significantly increased DUSP6 levels in the vessel wall. Compared with wild-type mice, *Dusp6*-deficient mice had smaller neointima. In vitro, IL-1β induced DUSP6 expression and increased VSMC proliferation and migration. Lack of DUSP6 reduced IL-1β-induced VSMC proliferation and migration. DUSP6 deficiency did not affect IL-1β-stimulated ERK1/2 activation. Instead, ERK1/2 inhibitor U0126 prevented DUSP6 induction by IL-1β, indicating that ERK1/2 functions upstream of DUSP6 to regulate DUSP6 expression in VSMCs rather than downstream as a DUSP6 substrate. IL-1β decreased the levels of cell cycle inhibitor p27 and cell–cell adhesion molecule N-cadherin in VSMCs, whereas lack of DUSP6 maintained their high levels, revealing novel functions of DUSP6 in regulating these two molecules. Taken together, our results indicate that lack of DUSP6 attenuated neointima formation following arterial injury by reducing VSMC proliferation and migration, which were likely mediated via maintaining p27 and N-cadherin levels.

## 1. Introduction

Cardiovascular diseases, encompassing ischemic heart disease and stroke, remain the leading cause of death worldwide, contributing to disability and rising healthcare costs [1]. Coronary heart disease—the leading cause of deaths attributable to cardiovascular disease in the United States—primarily results from vessel occlusion [2]. Revascularization procedures, such as coronary angioplasty and stent insertion, are vital to relieve occlusion of arteries; however, interventional procedures may injure arterial walls, leading to blood vessel re-occlusion and occurrence of in-stent restenosis [3]. The incidence of in-stent restenosis and the necessity for repetition of revascularization occur at a rate of 1–2% per year [4]. Vascular smooth muscle cells (VSMCs) are the predominant component of the arterial medial layer. Apart from a structural role, the main function of VSMCs in the blood vessels is to contract and regulate vascular tone and blood pressure [5]. In a normal vessel wall, VSMCs exhibit a differentiated, contractile phenotype and proliferate at an extremely low rate. In response to injury, VSMCs within the vessel wall rapidly dedifferentiate and change from a quiescent and contractile phenotype to a synthetic phenotype with enhanced proliferative and migratory properties and increased matrix production, contributing to lesion formation and occlusion of blood vessels [6,7,8]. Therefore, it is crucial to modulate VSMC proliferation and migration under pathological conditions to mitigate intimal hyperplasia.

The mitogen-activated protein kinase (MAPK) cascades transduce extracellular signals from different stimuli to elicit cellular responses [9]. Of the MAPK cascades, the ERK1/2 pathway regulates many cellular processes, affecting physiological functions [10]. ERK1/2 activation drives actin polymerization, adhesion turnover, and cell contraction for cell movement [11]. Genetic inhibition of cardiac ERK1/2 promotes stress-induced apoptosis and heart failure in vivo [12]. In VSMCs, loss of SM22α impairs angiotensin-II-induced ERK1/2 phosphorylation, concomitant with reduced vascular contraction [13]. Furthermore, MAPK pathway activation, particularly ERK1/2, is pivotal in mitogen-induced VSMC proliferation and migration [14]. In the presence of inflammatory cytokines, such as IL-1β, the activation of tyrosine kinase receptor leads to the phosphorylation of ERK1/2 [15], which is associated with activation of genes responsible for cell cycle progression and subsequent proliferation [16]. Collectively, ample evidence reveals the important functions of ERK1/2 pathway in cardiovascular disease [14,16]. As such, proper modulation of ERK1/2 signaling to maintain a balance between its activation and inactivation is critical.

Protein phosphatases negatively regulate MAPK signaling [17]. Dual-specificity phosphatases (DUSPs) are a subfamily of protein tyrosine phosphatases, which are able to dephosphorylate MAPKs [18]. Because of their role in modulating MAPK activity, DUSPs have been implicated as major modulators of critical signaling pathways, which are dysregulated in various diseases, including cardiac remodeling [19]. Dual-specificity phosphatase 6 (DUSP6, also named MKP3), a member of the MAPK phosphatase family, specifically dephosphorylates activated ERK1/2 [20]. DUSP6 interacts with ERK1/2 through a conserved binding motif, enabling it to dephosphorylate dual phosphates at threonine and tyrosine positions, thereby regulating the intensity of the ERK1/2 cascade [20]. Studies have revealed diverse functions of DUSP6 in different cellular contexts and diseases. DUSP6 can either promote or inhibit cellular proliferation. While DUSP6 induces proliferation in breast cancer, lung cancer, and thyroid carcinoma, DUSP6 inhibits proliferation and suppresses tumor progression in pancreatic, ovarian, and non-small lung cancer [21]. Interestingly, DUSP6 has been reported to participate in diet-induced obesity and dextran sulfate sodium-induced colitis [22,23,24]. Despite its involvement in various cellular processes and diseases, it is not clear whether DUSP6 plays a role in regulating VSMC function and vascular disease.

In this study, through utilization of an arterial denudation model in wild-type and *Dusp6* knockout (*Dusp6^−/−^*) mice, we uncovered a new function of DUSP6 within the realm of vascular injury. We showed that compared to wild-type mice, *Dusp6^−/−^* mice displayed reduced neointima formation. This reduction was attributed to the inhibition of VSMC proliferation and migration, mediated via modulation of cell cycle inhibitor p27 and cell–cell adhesion molecule N-cadherin levels by DUSP6.

## 2. Results

### 2.1. DUSP6 Is Increased in the Vessel Wall after Injury and Promotes VSMC Proliferation In Vitro

**Figure 1:**
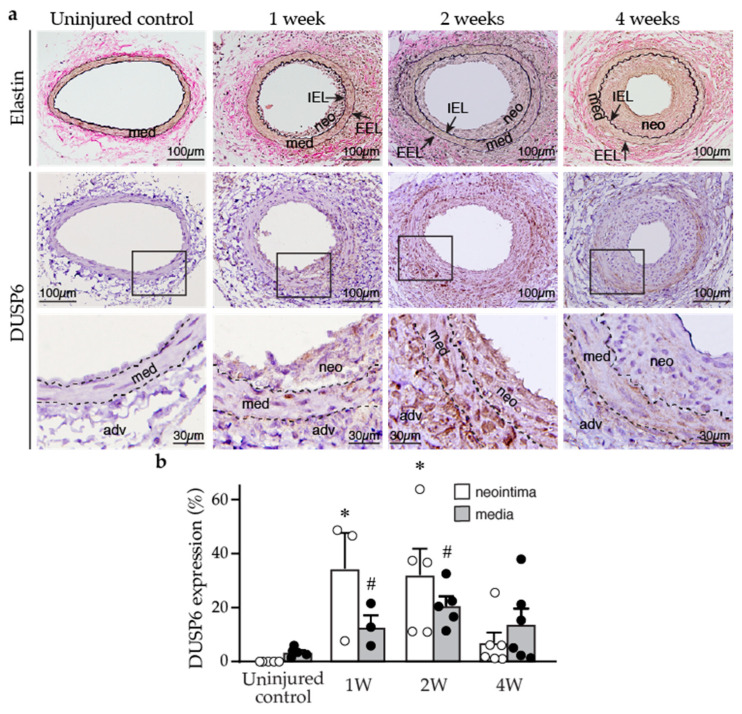
Arterial injury induces neointima formation and DUSP6 expression in mice. Wild-type mice were subjected to femoral artery injury, and vessels were harvested after 1, 2, and 4 weeks for histological analysis. (**a**) Verhoeff’s stain and immunostaining with DUSP6 antibody were performed on vessel sections to delineate elastin layers (top row) and DUSP6 expression (brown, middle row), respectively. Bottom row, higher magnification of the box in the middle row, respectively. adv, adventitia; IEL, internal elastic lamina; EEL, external elastic lamina; med, media; neo, neointima. Arrows indicate IEL and EEL, respectively. Dashed lines demarcate medial layer. (**b**) Quantitation of DUSP6 expression in the media and neointima of arteries after denudation injury. *n* = 3–6, * *p* < 0.05 vs. control neointima, # *p* < 0.05 vs. control media, one-way ANOVA followed by Tukey’s test.

To investigate the potential role of DUSP6 in the blood vessels, we first examined DUSP6 expression in the arteries of wild-type mice under normal physiological conditions and after femoral arterial injury. Immunostaining of arterial sections showed that baseline DUSP6 expression was low in the control uninjured mouse femoral arteries (Figure 1a). In comparison, DUSP6 expression was increased following arterial denudation injury in the vessel wall, including media and neointima, at 1, 2, and 4 weeks after injury (Figure 1a). Quantitative analysis showed that injury-increased DUSP6 expression was evident at 1 week, peaking at 2 weeks, and tapering off after 4 weeks (Figure 1b). Interestingly, the time period with most increases in DUSP6 in the arterial wall correlated with the period with most enhanced proliferative capacities of VSMCs after vascular injury [25]. These data indicate that DUSP6 is potentially involved in neointima formation after injury in mice.

**Figure 2:**
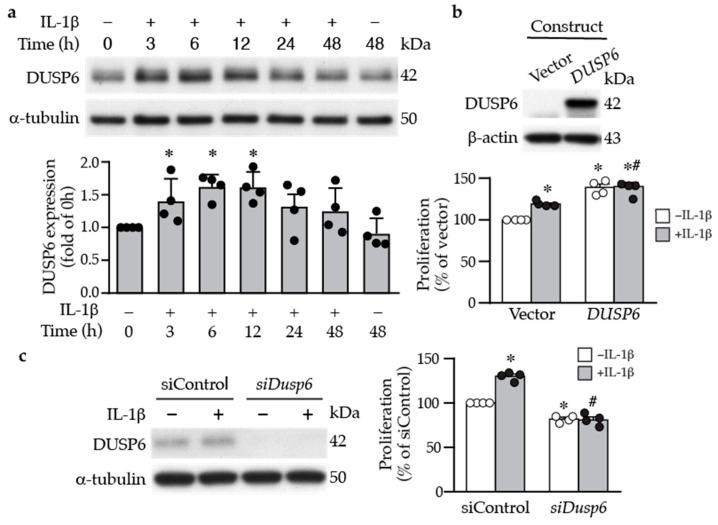
DUSP6 promotes VSMC proliferation. (**a**) Serum-starved VSMCs were treated with IL-1β (10 ng/mL), and DUSP6 expression was examined at different time points by Western blot analysis. α-Tubulin was detected as an internal control. A representative blot is shown (*n* = 4). Quantitation of DUSP6 levels in VSMCs. * *p* < 0.05 vs. control, Mann–Whitney U-test. (**b**) VSMCs were transfected with vector pCMV6-AN-3DDK or pCMV6-AN-3DDK-h *DUSP6* expression plasmid. Proteins were prepared after 24 h to examine DUSP6 expression by Western blotting (*n* = 4). Transfected cells were serum starved, treated with or without IL-1β for 24 h, and proliferation was assessed and normalized to vector without IL-1β (*n* = 4). * *p* < 0.05 vs. vector without IL-1β, # *p* < 0.05 vs. vector with IL-1β, Mann–Whitney U-test. (**c**) VSMCs were transfected with control siRNA or *Dusp6* siRNA and then treated with or without IL-1β for 24 h, and proteins were harvested for Western blotting for DUSP6 to assess knockdown efficiency. α-Tubulin was used as a loading control. Knockdown cells were serum starved, treated with or without IL-1β for 24 h, and proliferation was measured and normalized to vector without IL-1β (*n* = 4). * *p* < 0.05 vs. siControl without IL-1β, # *p* < 0.05 vs. siControl with IL-1β, Mann–Whitney U-test.

Inflammatory cytokines and growth factors are released into the injured sites to elicit proliferative and migratory responses of vascular cells following injury [26]. We thus examined the effects of inflammatory cytokines on DUSP6 expression in primary VSMCs. Under normal conditions, serum-starved VSMCs exhibited low baseline DUSP6 expression; in contrast, IL-1β increased DUSP6 level, peaking at 6–12 h (Figure 2a). To assess the role of DUSP6 in VSMC proliferation, we transfected VSMCs with vector or *Dusp6* expression plasmid (Figure 2b), treated with or without IL-1β, and then, proliferation was measured after 24 h. Indeed, overexpressing *Dusp6* increased VSMC proliferation even in the absence of IL-1β; *Dusp6* overexpression further enhanced IL-1β-induced proliferation (Figure 2b). Conversely, knocking down *Dusp6* using siRNA in VSMCs (Figure 2c) significantly reduced basal and IL-1β-induced proliferation compared to siControl (Figure 2c). These data indicate that DUSP6 promotes VSMC proliferation.

### 2.2. Dusp6 Deficiency Reduces Neointima Formation and Decreases VSMC Proliferation

**Figure 3:**
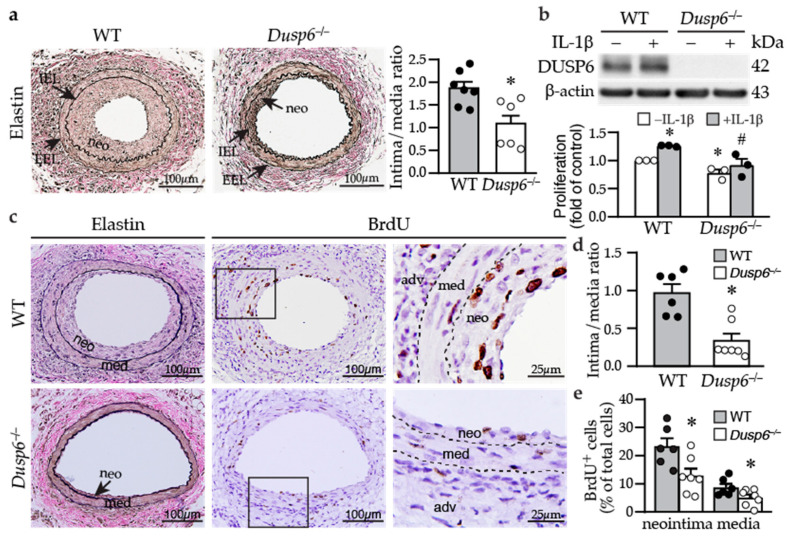
DUSP6 deficiency reduces neointima formation in response to arterial injury in mice. (**a**) Wild-type (WT) and *Dusp6^−/−^* mice were subjected to femoral artery injury. Vessels were harvested 4 weeks later for Verhoeff’s staining for elastin layers. IEL, internal elastic lamina; EEL, external elastic lamina; neo, neointima. Arrows indicate IEL and EEL, respectively. Quantitative morphometric analysis of intimal and medial area, expressed as intima/media ratio (WT, *n* = 7; *Dusp6^−/−^*, *n* = 6). * *p* < 0.05 vs. WT, two-tailed unpaired Student’s *t*-test. (**b**) Serum-starved wild-type and *Dusp6^−/−^* VSMCs were treated with or without IL-1β. DUSP6 expression and proliferation were assessed by Western blotting and BrdU incorporation assays, respectively (*n* = 3). * *p* < 0.05 vs. WT without IL-1β, # *p* < 0.05 vs. WT + IL-1β, Mann–Whitney U-test. (**c**) WT (*n* = 6) and *Dusp6^−/−^* mice (*n* = 7) were subjected to femoral artery injury, and vessels were harvested 2 weeks later. Mice were injected with BrdU at 16–18 h and 1–2 h before harvest. Vessel sections were stained with Verhoeff’s staining for elastin layers (left column) and with BrdU antibody (brown) for proliferating cells (middle column). Right column, higher magnification of the box in the middle column, respectively. Dashed lines demarcate medial layer. med, media; neo, neointima; adv, adventitia. (**d**) Quantitation of intima/media ratio of WT (*n* = 6) and *Dusp6^−/−^* (*n* = 7) mice 2 weeks after injury. * *p* < 0.05 vs. WT, two-tailed unpaired Student’s *t*-test. (**e**) BrdU-positive cells were quantified and expressed as % of total cells in the neointima or media (WT, *n* = 6; *Dusp6^−/−^*, *n* = 7). * *p* < 0.05 vs. WT, two-tailed unpaired Student’s *t*-test.

Based on the findings that DUSP6 increased VSMC proliferation, we hypothesized that the absence of DUSP6 may attenuate neointima formation after injury. Wild-type and *Dusp6*^−/−^ mice were subjected to guide wire injury, and femoral arteries were harvested 4 weeks later. Verhoeff’s elastin staining revealed large neointima in the injured wild-type mice with a high intima/media (I/M) ratio of 1.87 ± 0.14, while small neointima was observed in the *Dusp6^−/−^* mice with a lower I/M ratio of 1.09 ± 0.22 (Figure 3a). Supporting the notion that lack of DUSP6 decreased VSMC proliferation, compared with wild-type VSMCs, *Dusp6^−/−^* VSMCs had reduced cellular proliferation without IL-1β stimulation and abolished IL-1β-induced proliferation (Figure 3b). To confirm that *Dusp6* deficiency decreases VSMC proliferation in vivo, we assessed neointima formation 2 weeks after injury—a time point where VSMCs still actively proliferate [25]. Modest neointima was observed in wild-type injured vessel 2 weeks after injury (Figure 3c), while *Dusp6^−/−^* injured artery only had small neointima, with significantly reduced I/M ratio (Figure 3d). Interestingly, there were many BrdU-positive cells within the neointima and media of injured wild-type artery (Figure 3c). By contrast, only a few BrdU-positive cells were detected in *Dusp6^−/−^* injured artery (Figure 3c). The quantitation of BrdU-positive cells showed that *Dusp6* deficiency significantly attenuated BrdU incorporation into neointima and media, suggesting *Dusp6* deficiency decreased VSMC proliferation in the injured arterial wall (Figure 3e). The results from both in vitro and in vivo experiments indicate that DUSP6 plays a critical role in the response to vascular injury.

### 2.3. ERK1/2 Activation Precedes DUSP6 Induction in VSMCs following IL-1β Stimulation

**Figure 4:**
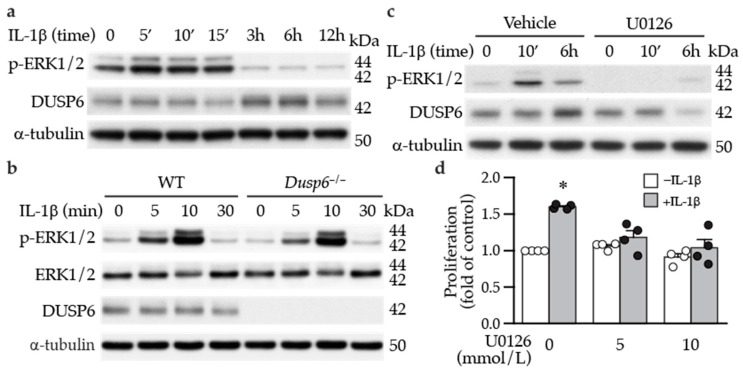
ERK1/2 activation precedes and mediates DUSP6 induction and VSMC proliferation by IL-1β. (**a**) Serum-starved VSMCs were treated with IL-1β, and total proteins were isolated at different time points for Western blotting to detect ERK1/2 activation and DUSP6 expression. As a loading control, blots were subsequently hybridized with α-tubulin antibody. (**b**) Serum-starved wild-type (WT) and *Dusp6^−/−^* VSMCs were treated with IL-1β, and proteins were prepared at different time points for Western blotting to detect phosphorylated and total ERK1/2 and DUSP6 expression. α-Tubulin was used as a loading control. *n* = 4. (**c**) Serum-starved VSMCs were pretreated with ERK1/2 inhibitor U0126 30 min prior to IL-1β treatment, and total proteins were isolated at different time points for Western blotting to detect ERK1/2 activation and DUSP6 expression. α-Tubulin was used as a loading control. *n* = 4. (**d**) Serum-starved WT VSMCs were pretreated with different concentrations of U0126 prior to IL-1β stimulation. Proliferation was measured 24 h later. * *p* < 0.05 vs. vehicle without IL-1β, *n* = 4 each, Mann–Whitney U-test.

Since ERK1/2 activation is involved in cellular proliferation, and given that IL-1β promoted VSMC proliferation, at least in part, via DUSP6 (Figure 2), we first examined whether IL-1β affects ERK1/2 activation. Indeed, IL-1β induced ERK1/2 phosphorylation at early time points, peaking at 5–15 min and returning to baseline at later time points (3–12 h) (Figure 4a). Interestingly, ERK1/2 activation preceded DUSP6 induction by IL-1β (Figure 4a). Given that ERK1/2 is a well-known substrate of DUSP6, we hypothesized that DUSP6 deficiency would sustain IL-1β-induced ERK1/2 phosphorylation. However, IL-1β induced ERK1/2 activation to a similar degree in both wild-type and *Dusp6^−/−^* VSMCs (Figure 4b and Supplementary Figure S1a). We then used U0126 to inhibit ERK1/2 phosphorylation. As expected, U0126 abrogated IL-1β-induced ERK1/2 activation (Figure 4c). Intriguingly, U0126 abolished not only ERK1/2 activation but also DUSP6 induction by IL-1β (Figure 4c and Supplementary Figure S1b). These results indicate that ERK1/2 functions upstream of DUSP6 in controlling DUSP6 expression rather than downstream as a dephosphorylation substrate of DUSP6. Moreover, inhibiting ERK1/2 activation with U0126 inhibited IL-1β-induced VSMC proliferation (Figure 4d), indicating that ERK1/2 activation is required for IL-1β to promote VSMC proliferation, likely through DUSP6 induction.

### 2.4. DUSP6 Regulates Cell Cycle Inhibitor p27 Levels

**Figure 5:**
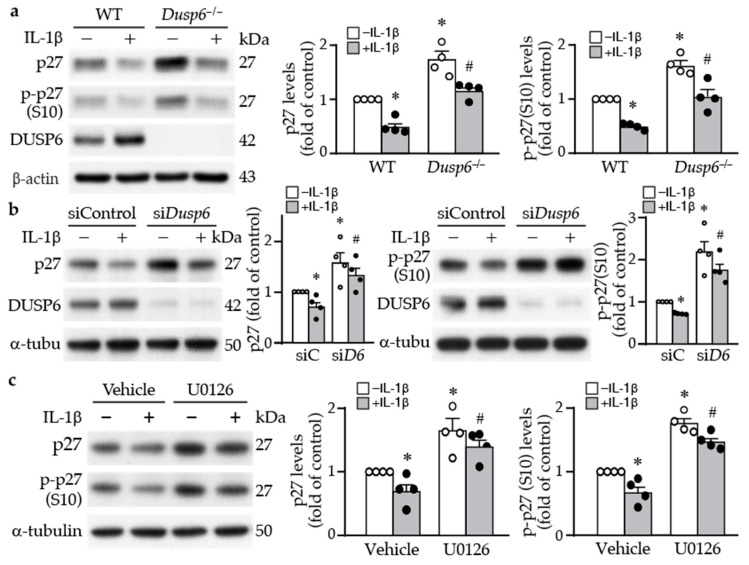
DUSP6 regulates cell cycle inhibitor p27 levels in VSMCs. (**a**) Serum-starved wild-type (WT) and *Dusp6^−/−^* VSMCs were stimulated with or without IL-1β for 12 h, and proteins were prepared for Western blotting to detect total p27, phospho-p27 at serine 10 (p-p27 (S10)), DUSP6, and β-actin as loading control. *n* = 4. Expression levels of total p27 and p-p27 were quantified. *n* = 4 each, * *p* < 0.05 vs. WT without IL-1β; # *p* < 0.05 vs. WT with IL-1β, Mann–Whitney U-test. (**b**) WT VSMCs were transfected with siControl (siC) or si*Dusp6* (si*D6*) prior to treatment with or without IL-1β. Total p27, p-p27, and α-tubulin (α-tubu) as loading control. Representative blot from *n* = 4 each. Protein levels were quantified. * *p* < 0.05 vs. siControl without IL-1β; # *p* < 0.05 vs. siControl with IL-1β, Mann–Whitney U-test. (**c**) Serum-starved VSMCs were pretreated with U0126 prior to IL-1β stimulation for 12 h, and proteins were harvested for Western blot analysis to detect total p27, p-p27 (S10), and α-tubulin as loading control. *n* = 4. Levels of p27 and p-p27 were quantified. *n* = 4 each, * *p* < 0.05 vs. vehicle without IL-1β; # *p* < 0.05 vs. vehicle with IL-1β, Mann–Whitney U-test.

To determine the molecular mechanism by which DUSP6 promotes VSMC proliferation, we examined proliferation-related proteins. Western blotting revealed that loss of DUSP6 increased the baseline levels of cell cycle inhibitor p27 and its phosphorylation at serine 10, which increases its protein stability [27] (Figure 5a). IL-1β decreased total and phospho-p27 (p-p27) levels in VSMCs of both genotypes; nevertheless, total and p-p27 levels were higher in *Dusp6*-deficient than in wild-type cells (Figure 5a), suggesting DUSP6 may regulate p27 levels. To confirm this finding, we used siRNA to knock down DUSP6 levels in VSMCs. Consistent with the finding in *Dusp6^−/−^* VSMCs, compared with siControl, knocking down DUSP6 expression increased p27 and p-p27 baseline levels, and even after IL-1β stimulation, their levels were higher than those of siControl (Figure 5b). Because inhibiting ERK1/2 activation by U0126 suppressed not only IL-1β-induced DUSP6 expression but also VSMC proliferation (Figure 4c,d), we then examined the effect of U0126 on p27 expression in VSMCs. Interestingly, U0126 enhanced total p27 and p-p27 levels in VSMCs, both at baseline and after IL-1β stimulation (Figure 5c). These results suggest that loss of DUSP6 reduces VSMC proliferation by increasing p27 levels.

### 2.5. Lack of DUSP6 Reduces VSMC Migration

**Figure 6:**
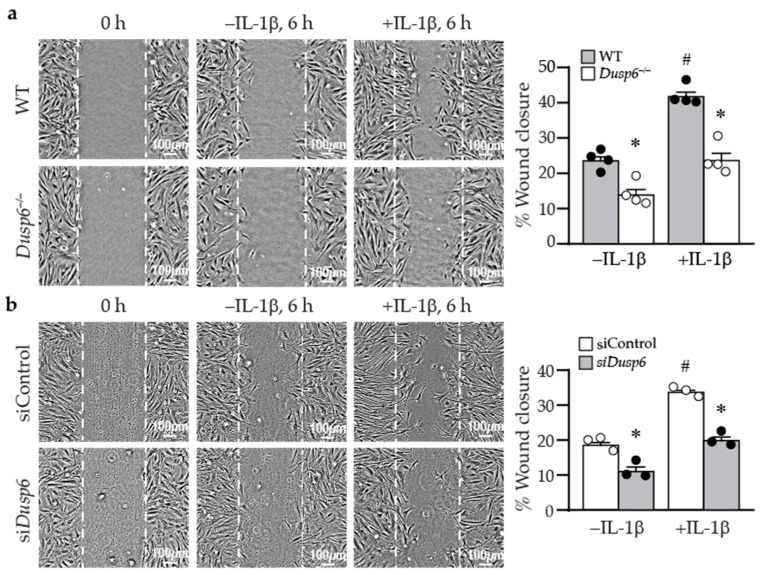
DUSP6 deficiency abrogates basal and IL-1β-induced VSMC migration. (**a**) WT and *Dusp6^−/−^* VSMCs were wounded and then stimulated with or without IL-1β (10 ng/mL) for migration assays, and wound closure was evaluated at 6 h after wounding. Dashed lines indicate initial wound margins. Quantitation of wound closure at 6 h (*n* = 4 each group). * *p* < 0.05 vs. respective WT, # *p* < 0.05 vs. WT without IL-1β, one-way ANOVA followed by Tukey’s test. (**b**) WT VSMCs were transfected with siControl or si*Dusp6*, wounded, stimulated with or without IL-1β, and wound closure was assessed. Quantitation of wound closure at 6 h (*n* = 3 each group). * *p* < 0.05 vs. siControl in each group, # *p* < 0.05 vs. siControl without IL-1β, one-way ANOVA followed by Tukey’s test.

In addition to proliferation, VSMC migration plays a pivotal role in developing intimal hyperplasia after vascular injury. We next examined whether DUSP6 has a role in cellular migration using wound healing assays. At time 0, wild-type and *Dusp6^−/−^* VSMCs had similar wounds (Figure 6a). In the absence of IL-1β, there was a basal migratory ability of cells to migrate toward the wound margin after 6 h; nevertheless, fewer *Dusp6^−/−^* VSMCs migrated toward the wound margin (Figure 6a). Although IL-1β induced cellular migration in both wild-type and *Dusp6^−/−^* VSMCs, DUSP6 deletion significantly reduced IL-1β-induced migration (Figure 6a). Knocking down DUSP6 with siRNA significantly abrogated VSMC migration in the absence or presence of IL-1β (Figure 6b). These results indicate that lack of DUSP6 in VSMCs reduces baseline and IL-1β-induced cellular migration.

**Figure 7:**
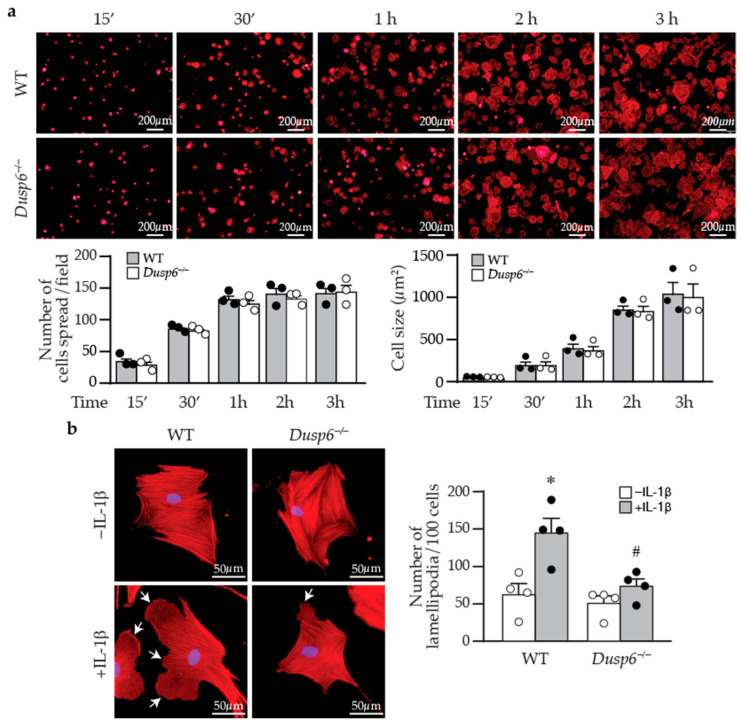
DUSP6 deficiency inhibits lamellipodia formation after IL-1β stimulation in VSMCs. (**a**) Wild-type or *Dusp6^−/−^* VSMCs were plated, fixed, and stained with phalloidin (red) at indicated time points. Images were captured with florescence microscope Olympus IX71, 40× magnification; representative images from three independent experiments are shown. The number of cells attached and spread per field was quantified. Cell size was measured with ImageJ and expressed as µm^2^/cell. (**b**) WT and *Dusp6^−/−^* VSMCs were serum starved prior to stimulation with or without IL-1β for 60 min. Phalloidin staining (red) was performed to assess lamellipodia formation. Arrows indicate lamellipodia. Lamellipodia were counted in 100 cells and expressed as the number of lamellipodia per 100 cells. *n* = 4 independent experiments. * *p* < 0.05 vs. WT without IL-1β, # *p* < 0.05 vs. WT with IL-1β, one-way ANOVA followed by Sidak’s test.

Cell attachment to and spreading on the matrix affect cellular migration [28]. We thus examined whether DUSP6 affects these processes. Phalloidin staining showed that wild-type cells were almost 100% attached within 2–3 h after plating; DUSP6 deletion did not affect VSMC attachment and spreading or cell size compared with wild-type cells at various time points following plating (Figure 7a). As cellular migration requires focal adhesion assembly and disassembly processes to attach and detach from the matrix [28], we next examined focal adhesion complex assembly and disassembly. Immunostaining with paxillin, p-FAKY397, and p-FAKY861 in wild-type and *Dusp6^−/−^* VSMCs showed that DUSP6 deficiency did not affect focal adhesion assembly and disassembly in VSMCs. Interestingly, we observed that after IL-1β stimulation, in comparison with wild-type cells, *Dusp6^−/−^* VSMCs had reduced lamellipodia formation with decreased size and number (Figure 7b). These results suggest that the reduction in IL-1β-induced migration of *Dusp6^−/−^* VSMCs may be due in part to decreased lamellipodia formation, rather than differences in adhesion and spreading.

### 2.6. DUSP6 Deficiency Increases Cell–Cell Adhesion Molecule N-cadherin in VSMCs

**Figure 8:**
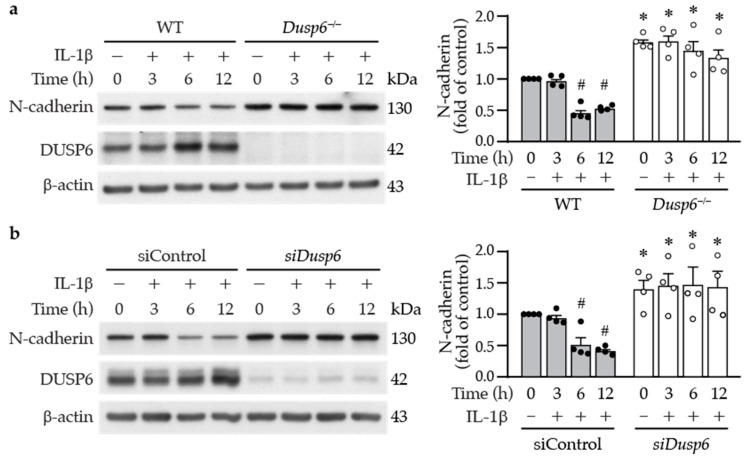
Lack of DUSP6 increases cell–cell adhesion molecule N-cadherin in VSMCs. (**a**) Serum-starved WT or *Dusp6^−/−^* VSMCs were treated with IL-1β for different time periods, and proteins were prepared for Western blotting to detect N-cadherin, DUSP6, and β-actin as loading control. N-cadherin levels were quantified, *n* = 4. * *p* < 0.05 vs. WT at respective time points, # *p* < 0.05 vs. WT without IL-1β at 0 h, Mann–Whitney U-test. (**b**) WT VSMCs were transfected with siControl or si*Dusp6*, serum starved, treated with IL-1β, and then analyzed as in (**a**). N-cadherin levels were quantitated. *n* = 4. * *p* < 0.05 vs. siControl at each time point, # *p* < 0.05 vs. siControl without IL-1β at 0 h, Mann–Whitney U-test.

In the wound healing assays, we observed in the live cell imaging video that, in contrast with wild-type cells, the neighboring *Dusp6^−/−^* VSMCs appeared to be adhered together and migrated slower toward the wound (Supplementary Video S1). N-cadherin is the most abundant cell–cell adhesion molecule [29], and its downregulation in the neointima has been reported to stimulate VSMC migration [30]. We therefore wondered whether N-cadherin participates in DUSP6-regulated VSMC migration. Western blotting showed that N-cadherin was expressed in wild-type VSMCs; intriguingly, the loss of DUSP6 resulted in significant increases in N-cadherin levels (Figure 8a), suggesting a potential involvement of N-cadherin in the reduced migration of *Dusp6^−/−^* VSMCs. To further evaluate this possibility, we treated VSMCs with IL-1β and examined N-cadherin expression at different time points. IL-1β decreased N-cadherin levels at 6 and 12 h (Figure 8a); the time points coincided with induction of DUSP6 (Figure 2a). Importantly, DUSP6 deficiency exhibited higher baseline levels of N-cadherin; furthermore, IL-1β did not decrease N-cadherin levels at 6 or 12 h time points (Figure 8a). The cadherin–catenin complex has been proposed to regulate VSMC behavior [31]; we thus examined β-catenin expression. Western blot analysis showed no differences in β-catenin expression levels between wild-type and *Dusp6^−/−^* VSMCs, either at baseline or after IL-1β stimulation (Supplementary Figure S2), suggesting that DUSP6 may not regulate β-catenin in VSMCs. As *Dusp6* knockdown impaired the migratory capacity of VSMCs (Figure 6b), we then examined the effect of knocking down *Dusp6* expression on N-cadherin levels in VSMCs. Intriguingly, *Dusp6* knockdown in VSMCs increased baseline N-cadherin level and prevented IL-1β-reduced N-cadherin levels at 6 and 12 h time points (Figure 8b). Together, these results indicate that DUSP6 potentially regulates N-cadherin levels.

## 3. Discussion

In this study, we discovered that in VSMCs, the baseline levels of the cell cycle inhibitor p27 and the cell–cell adhesion molecule N-cadherin were higher in the absence of DUSP6. Therefore, despite p27 and N-cadherin were downregulaed by IL-1β, their levels in *Dusp6*-deficient VSMCs were still higher than wild-type cells under inflammatory conditions, resulting in reduced proliferation and migration of VSMCs and reduced neointima formation after vascular injury. Our study unveiled a novel role of DUSP6 in regulating vascular disease.

We found that DUSP6 did not modulate ERK1/2 activation in VSMCs, as lack of DUSP6 did not increase or sustain IL-1β-induced ERK1/2 phosphorylation levels (Figure 4b). This finding is in contrast to a study, which showed that peripheral phosphorylated ERK1/2 levels in MKP3 (*Dusp6*) knockout mice remained elevated after injury [32] and the general belief that DUSP6 is a negative regulator of ERK1/2 activation [33]. Interestingly, the result where IL-1β-induced ERK1/2 phosphorylation in VSMCs was not affected by DUSP6 is similar to a report showing that ERK1/2 activation is not increased or prolonged in *Dusp6^−/−^* mouse embryo fibroblasts after stimulation with phenylephrine, although it affects baseline phosphorylated ERK1/2 levels [34]. Another interesting finding is that IL-1β-induced DUSP6 expression was mediated by ERK1/2 activation (Figure 4c), indicating that ERK1/2 functions upstream of DUSP6 to induce its levels rather than as a substrate of DUSP6 in VSMCs under an inflammatory condition. This notion is supported by a previous study showing that DUSP6 is inducible by fibroblast growth factor (FGF) signaling via ERK1/2 activity in NIH 3T3 cells [35]. Although Ekerot et al. proposed that DUSP6 acts as a negative regulator of ERK activity to exert negative feedback regulation of FGF signaling [35], our results in *Dusp6^−/−^* VSMCs clearly demonstrated that lack of DUSP6 did not affect IL-1β-ERK1/2 signaling axis and did not serve a negative feedback function, which differed from the proposition by Ekerot et al. Together, it is conceivable that in VSMCs, additional DUSPs, other than DUSP6, which have the capacity to dephosphorylate ERK1/2, are responsible for the inactivation of ERK1/2 [36,37], particularly under pathological conditions. Alternatively, DUSP6 may have phosphatase activity independent functions in VSMCs. Supporting this notion, DUSP6 was found to promote endothelial inflammation independent of ERK1/2 signaling [38].

DUSP6 deficiency in VSMCs led to an increase in total and phospho-p27 at baseline levels, maintaining them at high levels even after IL-1β stimulation (Figure 5a). p27 is one of the cyclin-dependent kinase inhibitors (CDKs), which control cell cycle progression [39]. Serine 10 phosphorylation of p27 increases its protein stability [27]. The increased p27 level was reflected functionally in decreased VSMC proliferation in the knockout or knockdown of *Dusp6* in VSMCs (Figure 2c and Figure 5a,b). The inhibition of VSMC proliferation has been attributed to inhibition of CDKs by p27 via preventing the progression of G1 to the S phase of the cell cycle, ultimately attenuating intimal hyperplasia following arterial injury [40,41]. Transcription factor FKHRL1 or mediators such as baicalin, which increase p27 levels in VSMCs, prevent injury-induced neointimal hyperplasia [42,43]. Our finding adds DUSP6 to the list of molecules regulating p27 levels in VSMCs. This is likely the mechanism by which DUSP6 deficiency resulted in reduced proliferative capacity in response to IL-1β stimulation, contributing, at least in part, to mitigating neointima formation in mice after injury.

The live cell imaging of the wound healing experiments revealed a distinctive behavior in neighboring *Dusp6^−/−^* VSMCs compared with wild-type cells. Notably, these *Dusp6^−/−^* VSMCs exhibited a tendency to adhere to each other, and their migration toward the wound site was notably slower than that observed in wild-type cells (Supplementary Video S1). This behavior suggests enhanced cell–cell adhesion in *Dusp6^−/−^* VSMC compared with wild-type cells. Indeed, *Dusp6^−/−^* VSMCs exhibited higher levels of N-cadherin at baseline, and DUSP6 deficiency mitigated IL-1β-reduced N-cadherin levels (Figure 8). This result suggests that DUSP6 plays a regulatory role in VSMC migration through the N-cadherin axis. Concordant with our results, downregulation of N-cadherin in VSMCs has been demonstrated to induce neointima formation in a porcine restenosis model, potentially through RhoA deactivation toward enhancing VSMC migration capacity [44]. Mechanistically, during migration, N-cadherin translocates to the posterior-lateral cell edge of VSMCs, and the cell–cell adhesions via N-cadherin determine polarity and consequently decrease migratory capacity [45]. Nevertheless, contradictory results have been reported in some studies. N-cadherin inhibition with antagonist, neutralizing antibodies and adenoviral expression of dominant negative N-cadherin reduced VSMC migration and intimal thickening [46]. The role of N-cadherin in VSMCs remains controversial. E-cadherin is a member of the cadherin family, and its role in VSMCs remains unclear. Further investigations of N-cadherin and E-cadherin are needed to clarify their roles in VSMCs.

**Figure 9:**
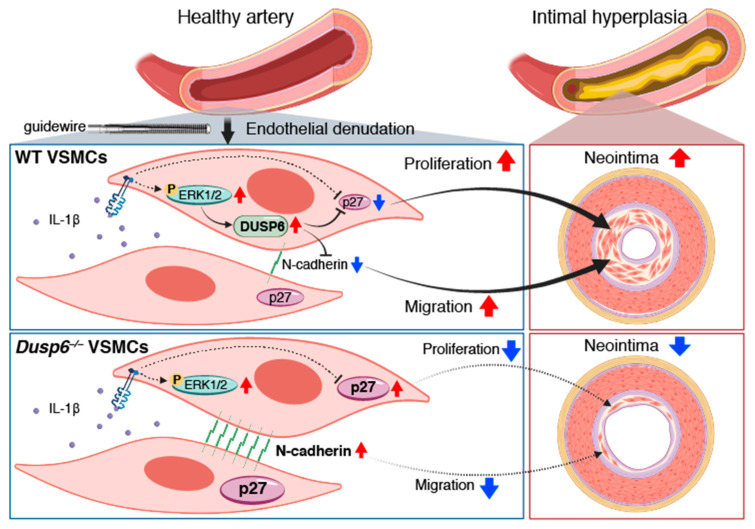
DUSP6 deficiency attenuates arterial-injury-induced intimal hyperplasia in mice. Arterial injury, such as guide wire denudation of endothelium, releases inflammatory cytokines, including IL-1β, at the injured site. IL-1β activates ERK1/2 pathways, which induces DUSP6 expression in VSMCs, together with yet to be identified molecules/mediators to decrease p27 and N-cadherin levels, leading to enhanced cellular proliferation and migration, and eventual intimal hyperplasia after injury. On the other hand, lack of DUSP6 increases baseline levels of p27 and N-cadherin in VSMCs. Furthermore, lack of DUSP6 maintains high levels of p27 and N-cadherin, resulting in reduced VSMC proliferation and migration, and consequently mitigating neointima formation. Red arrows indicate upregulation while blue arrows indicate downregulation. Created with Biorender.com.

In summary, our study identified a novel role of DUSP6 in VSMCs and vascular injury (Figure 9). Inflammatory cytokine IL-1β activates the ERK1/2 pathway to induce DUSP6 expression, together with yet to be identified molecules/mediators, to downregulate CDK inhibitor p27 and cell–cell adhesion molecule N-cadherin, consequently promoting VSMC proliferation and migration and subsequent neointima formation and restenosis after arterial injury. On the other hand, the absence of DUSP6 increases baseline levels of p27 and N-cadherin. Furthermore, lack of DUSP6 maintains high levels of p27 and N-cadherin even under inflammatory conditions, leading to decreased VSMC proliferation and migration, and ultimately mitigating arterial-injury-induced intimal hyperplasia. Our findings propose DUSP6 as a potential target for the development of therapeutic strategies for occlusive vascular disease.

## 4. Materials and Methods

### 4.1. Animal Care and Genotyping

All mice were maintained at a National Health Research Institutes (NHRI) animal facility in Taiwan (AAALAC accredited #001596, 12 h light–dark cycle). Mice were housed in a ventilated transparent plastic cage (3–4 mice/cage; Bio-Zone, 410 cm^2^ enclosure size, Lignocel FS-14 bedding). Water and food were provided ad libitum. Wild-type mice in C57BL/6 background were obtained from the National Laboratory Animal Center (Taipei, Taiwan) and acclimated for 2 weeks before any experiments. *Dusp6*-deficient (*Dusp6^−/−^*) mice in C57BL/6 background [24] were genotyped as described in the JAX Protocol.

### 4.2. Experimental Mouse Model of Femoral Artery Denudation Injury

All experimental procedures were performed in accordance with NIH guidelines (Guide for the care and use of laboratory animals) and approved by the Institutional Animal Care and Use Committee of the National Health Research Institutes, Zhunan, Taiwan (#NHRI-IACUC-107063-A). Animal studies are reported in compliance with the ARRIVE guidelines. Approximately 10–12-week-old male mice were subjected to the neointimal hyperplasia model of femoral artery endoluminal injury, as described [47]. Mice were anesthetized with tribromoethanol solution (Sigma-Aldrich, St. Louis, MO, USA, #T84802; 250 mg/kg) via intraperitoneal injection to achieve proper sedation. Endoluminal injury to the left common femoral artery was then performed with 3–5 passages of a hydrophilic coating guide wire (Abbott, Chicago, IL, USA, Hi-Torque Cross-it 100XT, 0.014” diameter) to denude endothelium. Mice were placed on a heating pad (low–medium heat) until recovery and then housed in individual cages.

### 4.3. Histological Analysis and Immunohistochemistry

At the indicated time points after injury, mice were sacrificed with an overdose of tribromoethanol solution (500–750 mg/kg) via intraperitoneal injection until they no longer displayed a withdrawal reflex in the hind limbs, perfused with saline, followed by 10% formalin (Sigma-Aldrich, #HT501128). In some experiments, to determine cellular proliferation in the injured vessel wall, mice were injected twice with bromodeoxyuridine (BrdU, Sigma-Aldrich) at 16–18 h and 1–2 h before harvest. The contralateral uninjured right femoral artery and injured left femoral artery were carefully dissected, further fixed in 10% formalin at 4 °C overnight, processed, and embedded in paraffin for histological analysis. Serial 4 μm cross-sections of the femoral arteries were collected. H&E staining on vessel sections was initially performed to assess morphology, and Verhoeff’s staining (Sigma) was used to delineate elastin layers. Three sets of cross-sections at 150 μm intervals were used for morphometry using NIH ImageJ software 1.53i. The intimal and medial areas were measured, and the intima-to-media ratio was calculated essentially as described [48].

For immunohistochemistry, sections were rehydrated, antigen retrieved, blocked with 5% bovine serum albumin, and incubated with primary antibody. To determine DUSP6 expression, sections were incubated with DUSP6 antibody (Abcam ab76310, 1:50, Cambridge, UK) overnight at 4 °C and counterstained with hematoxylin before mounting. DUSP6 expression levels were quantified by colorimetric analysis with ImageJ software 1.53i and expressed as a percentage of positive area in the neointima or media. To assess in vivo cell proliferation after injury, sections were stained with a monoclonal BrdU antibody (Dako #M0744, 1:100, Santa Clara, CA, USA) and counterstained with hematoxylin. The BrdU-positive cells were quantified and expressed as a percentage of total cells in neointima or media, respectively.

### 4.4. Primary VSMC Culture, IL-1β Stimulation, and Proliferation Assays

Primary VSMCs were isolated by enzyme digestion from wild-type and *Dusp6^−/−^* mouse aortas, and cells of passage 5 to 8 were used for experiments [49]. VSMCs were serum starved in DMEM containing 0.2% FBS for 24 h before stimulation with IL-1β (PeproTech, 10 ng/mL, Cranbury, NJ, USA) for different periods of time. Total proteins were then prepared for Western blot analysis to detect DUSP6 expression levels. To measure VSMC proliferation, cells were plated in a 24-well plate (8 × 10^3^ cells/well), serum starved in 0.2% FBS quiescent medium for 24 h, stimulated with or without IL-1β for 24 h, and cellular proliferation was assessed using BrdU cell proliferation kit (Millipore, QIA58, Burlington, MA, USA) or cell counting kit-8 (CCK-8) (Dojindo, CK04-05, Beijing, China), according to the manufacturer’s instructions.

### 4.5. Wound Healing Assays

Wound healing assays were performed to measure VSMC migratory capacity. Wild-type and *Dusp6^−/−^* VSMCs (2.5 × 10^5^ cells/well) were plated onto a 6-well dish, serum starved, and treated with 10 ng/mL mitomycin C (Roche, #10107409001, Basel, Switzerland) for 2 h to arrest cell growth prior to wounding with a p200 tip. IL-1β (0 or 10 ng/mL) was then used to induce cell migration. Wound images were captured with a microscope (Olympus CKX53, 40× magnification, Tokyo, Japan) at time 0 and at 6 h after IL-1β stimulation. The area of wound closure was quantified by ImageJ software 1.53i from 3 fields of each treatment and divided by the wound area at time 0, and it was presented as percent wound closure. To confirm the effects of DUSP6 on VSMC migration, *Dusp6* expression was knocked down with siRNA in wild-type VSMCs, and wound healing assays were then performed.

### 4.6. Western Blot Analysis

Total proteins were prepared from VSMCs [50] without stimulation or stimulated with IL-1β. Western blotting was then performed to detect the protein of interest. The primary antibodies used were DUSP6 (Abcam, ab76310), phospho-p27 S10 (Abcam, ab62364), p27 (Santa Cruz, sc-1641, Santa Cruz, CA, USA), phospho-Erk-1/2 Thr202/Tyr204 (Cell Signaling, #9101, Danvers, MA, USA), Erk-1/2 (Cell Signaling, #4696), N-cadherin (Cell Signaling, #13116), and β-catenin (BD Biosciences, #610154, San Diego, CA, USA). To verify equivalent loading, the blots were subsequently probed with α-tubulin (GeneTex, GTX112141, Irvine, CA, USA) or β-actin antibodies (Sigma-Aldrich, MAB1501, St. Louis, MO, USA).

### 4.7. DUSP6 Overexpression and Knockdown in VSMCs

To overexpress DUSP6 in VSMCs, wild-type VSMCs were electroporated with vector control (pCMV6-AN-3DDK) or expression plasmid pCMV6-AN-3DDK-hDUSP6 (8 µg DNA/1 × 10^6^ cells) with Gene Pulser Xcell™ Electroporation System (Bio-Rad, Hercules, CA, USA) using 400 V, 10 ms, square wave parameters. Transfected VSMCs were then recovered overnight in 10% FBS medium and starved for 24–48 h prior to IL-1β stimulation. Twenty-four hours later, total proteins were prepared for cell proliferation assessment or Western blot analysis.

To knock down DUSP6 expression in wild-type VSMCs, ON-TARGET plus SMART pool mouse DUSP6 small interfering RNA (siRNA) (Dharmacon, #L-040050-00, Lafayette, CO, USA) and negative control siRNA (Dharmacon, #D-001810-0X) were used. VSMCs were transfected with 20 nmol/L siRNA in Opti-MEM^®^ I Reduced Serum Medium (Thermo, #31985062, Waltham, MA, USA) using Lipofectamine RNAiMAX (Thermo, #13778150), as described by the manufacturer. Following overnight recovery, cells were starved and stimulated with or without IL-1β for 24 h. Proliferation (BrdU incorporation) or migration assays and Western blot analysis were then performed.

### 4.8. ERK1/2 Signaling and Cell Cycle Inhibitor p27

To evaluate ERK1/2 activation, wild-type and *Dusp6^−/−^* VSMCs were serum starved, treated with IL-1β, and total proteins were harvested at different time points for Western blotting to detect phospho- and total ERK1/2 and DUSP6. To inhibit ERK1/2 activation, serum-starved cells were pretreated with U0126 (10 µmole/L, Merk Millipore, Darmstadt, Germany) 30 min before stimulation with IL-1β, and total proteins were harvested at different time points for Western blotting. To assess the effect of ERK1/2 on VSMC proliferation, cells were pretreated with U0126 (0, 5, or 10 µmole/L) before stimulation with or without IL-1β for 24 h, and BrdU incorporation assays were performed to measure proliferation. To detect cell cycle inhibitor p27, starved VSMCs were treated with IL-1β for 12 h, and proteins were prepared for Western blotting to detect total p27 (Santa Cruz, sc-1641) and phospho-p27(S10) (Abcam, ab62364).

### 4.9. Cell Adhesion, Spreading, and Lamellipodia Formation

To assess VSMC adhesion and spreading, wild-type and *Dusp6^−/−^* VSMCs were seeded into μ-Slide VI 0.4 (ibidi, #80601) and allowed to attach for different periods of time. Adhered cells were fixed with 4% paraformaldehyde at the indicated time after plating (15 min, 30 min, 1 h, 2 h, and 3 h) for 10 min, permeabilized in 0.1% Triton-X for 5 min, blocked with 1% BSA for 1 h, and incubated with phalloidin (Thermo Alexa Fluor™ 594, Thermo, Waltham, MA, USA, 1:5000) for 30 min. Cells were then counterstained with DAPI (Invitrogen, #R37606, 1:5000, Waltham, MA, USA) for 5 min and mounted with 0.5% mounting buffer ddH_2_O (DAKO, S3023). Images were captured with a fluorescence microscope (Olympus IX71, 40× magnification). The percentage of attached/spread cells was calculated as the number of spread cells divided by total cell number (as determined by the number of blue nuclei) and multiplied by 100. The cell size of attached cells was measured with ImageJ software 1.53i. To assess lamellipodia formation, wild-type and *Dusp6^−/−^* VSMCs were seeded into ibidi μ-Slide VI 0.4 and serum starved for 24 h before stimulation with or without IL-1β for 1 h. Cells were then processed and stained with phalloidin as above. The number of lamellipodia was counted in 100 wild-type or *Dusp6^−/−^* VSMCs per experiment.

### 4.10. Statistical Analysis

Data were expressed as mean ± standard error of the mean (SEM) and derived from a minimum of 3 independent experiments. Parametric data were analyzed using two-tailed unpaired Student’s *t*-test or one-way ANOVA followed by Sidak or Tukey tests for multiple comparisons. These analyses encompassed the intima/media (I/M) ratio, BrdU-positive cells, DUSP6 expression, wound closure areas, lamellipodia number, cell spreading, and cell size. Non-parametric data, including protein blot levels, were analyzed using the Mann–Whitney U-test or one-way ANOVA followed by the Kruskal–Wallis test. Significance was set at *p* < 0.05. All analyses were conducted using GraphPad Prism 8 software.

## Data Availability

Raw data supporting the conclusions of this publication will be made available by the authors without undue reservations.

## Supplementary Materials

The following supporting information can be downloaded at: https://www.mdpi.com/article/10.3390/ijms242417136/s1.
