# Supplementary material for: Dual-Specificity Phosphatase 6 Deficiency Attenuates Arterial-Injury-Induced Intimal Hyperplasia in Mice
Source: Int J Mol Sci. 2023 Dec 5;24(24):17136. doi: 10.3390/ijms242417136 (PMC10742470; doi:10.3390/ijms242417136)
Supplement: Supplementary file 1 [file ijms-24-17136-s001.zip › IJMS Supplementary Materials.pdf]

Supplementary Materials

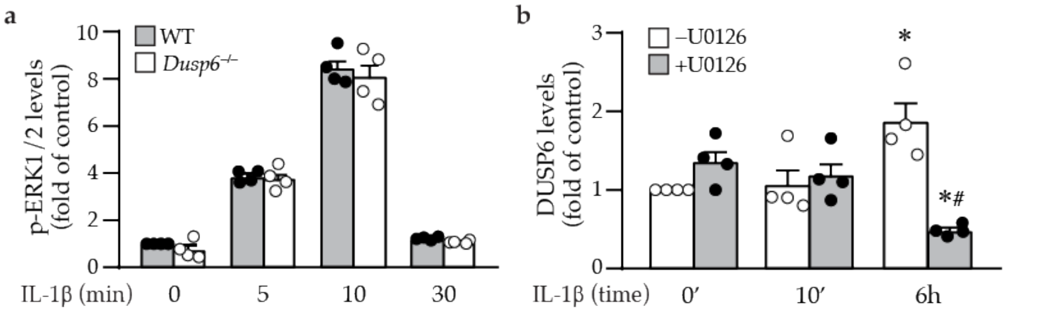

Figure S1. Quantitative analysis of IL-1β-induced ERK1/2 activation and DUSP6 expression in VSMCs. (**a**) Quantification of IL-1β-induced ERK1/2 phosphorylation at different time points after IL-1β stimulation in wild-type (WT) and *Dusp6*$^{-/-}$ VSMCs. n = 4 each. (**b**) Wild-type VSMCs were pretreated with or without U0126, an ERK1/2 inhibitor, prior to stimulation with or without IL-1β for the indicated time, and DUSP6 expression levels were quantified. n=4 each. **p* < 0.05 vs. vehicle (-U0126) without IL-1β treatment; #*p* < 0.05 vs. vehicle after IL-1β treatment for 6 h. Mann-Whitney U Test.

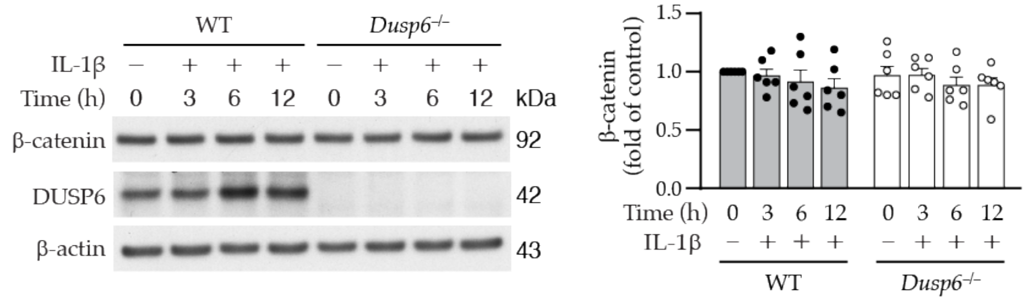

Figure S2. DUSP6 does not affect β-catenin levels. Serum-starved wild-type (WT) and *Dusp6*$^{-/-}$ VSMCs were treated with IL-1β (10 ng/mL) for the indicated times and proteins collected. Western blot analyis was performed to detect β-catenin and DUSP6 expression. β-Actin was used as an internal control. A representative of 6 independent experiments is shown. Levels of β-catenin were quantified. Expression level of WT cells at time 0 without IL-1β stimulation was set as 1. n=6 each. No significant difference of β-catenin level was detected at different time points between WT and *Dusp6*$^{-/-}$ VSMCs, Mann-Whitney U test.
